# 

**Published:** 1863-09

**Authors:** 


### BOOKS AND PAMPHLETS RECEIVED.

*A Manual of Instructions for Enlisting and Discharging Soldiers, with special reference to the Medical Examination of Recruits, and the detection of disqualifying and feigned diseases. By* ROBERT BARTHOLOW, A. M., M. D., *Assistant Surgeon U. S. Army, Surgeon in charge of McDougall General Hospital, Professor of Mil. Med. Jurisprudence, Army Medical School. Adopted by the Surgeon-General for issue to Medical Officers of the Army. Philadelphia:* J. B. LIPPINCOTT & Co., 1863. *For sale by Breed, Butler & Co.*

---

*What is the Modus Operandi of Medicines? Do they produce their effects by their Action on the Blood, as taught by all Modern Physiologists? or, do they produce their effects by their Action on the Organic Nervous System, through the agency of the Blood? The latter question considered and affirmatively answered. By* JOHN O'REILLY, M. D., F. R. C. S. I.

---

*On Artificial Dilatation of the Os and Cervix Uteri by Fluid Pressure from above; a reply to Drs. Keiller of Edinburgh, and Arnott and Barnes of London. By* HORATIO R. STORER, M. D., *of Boston, Surgeon to the Pleasant St. Hospital for Women, Member of the Obstetric and Medico-Chirurgical Societies of Edinburgh, etc. Re-printed from "The Boston Medical and Surgical Journal," for July* 2, 1862.

---

*Annual Announcement of the Jefferson Medical College of Philadelphia. Session of* 1863–4 *commences Monday, October* 12*th.*

*Lindsay & Blakiston's Physician's Visiting List, Diary, and Book of Engagements.*

The Publishers have been compelled, on account of the greatly increased cost of paper and other materials used in making THE VISITING LIST, to advance the price slightly over that of former years. The improvements added to the book, however, will, they trust, be regarded by the Profession as a full compensation for this necessary change.

*Price.*—Prepared for 25 patients, plain, 63 cents, tucks $1. For 50 patients, plain, 75 cents, tucks $1.25. For 100 patients, tucks, $2. For 100 patients in 2 vols., Jan'y to June and July to Dec. $2.50.

